# Changes in Pest Infestation Levels, Self-Reported Pesticide Use, and Permethrin Exposure during Pregnancy after the 2000–2001 U.S. Environmental Protection Agency Restriction of Organophosphates

**DOI:** 10.1289/ehp.11367

**Published:** 2008-08-15

**Authors:** Megan K. Williams, Andrew Rundle, Darrell Holmes, Marilyn Reyes, Lori A. Hoepner, Dana B. Barr, David E. Camann, Frederica P. Perera, Robin M. Whyatt

**Affiliations:** 1 Columbia Center for Children’s Environmental Health, Mailman School of Public Health, Columbia University, New York, New York, USA; 2 Centers for Disease Control and Prevention, Atlanta, Georgia, USA; 3 Southwest Research Institute, San Antonio, Texas, USA

**Keywords:** indoor air, insecticides, pregnancy, residential

## Abstract

**Background:**

Widespread residential pesticide use throughout the United States has resulted in ubiquitous, low-level pesticide exposure. The mix of active pesticide ingredients is changing in response to 2000–2001 regulations restricting use of the organophosphorus insecticides chlorpyrifos and diazinon.

**Objectives:**

We aimed to determine the impact of U.S. Environmental Protection Agency regulations on pest infestation levels, pesticide use, and pesticides measured in indoor air samples. METHODOLOGY: 511 pregnant women from innercity New York were enrolled between 2000 and 2006. Permethrin, a pyrethroid insecticide; piperonyl butoxide (PBO), a pyrethroid synergist; chlorpyrifos; and diazinon were measured in 48-hr prenatal personal air samples. Data on pest infestation and pesticide use were collected via questionnaire.

**Results:**

Eighty-eight percent of women reported using pesticides during pregnancy; 55% reported using higher-exposure pesticide applications (spray cans, pest bombs and/or professional pesticide applicators). Self-reported pest sightings and use of higher-exposure applications increased significantly after the regulations were implemented (*p* < 0.001). PBO, *cis*-, and *trans*-permethrin were detected in 75, 19, and 18% of personal air samples, respectively. Detection frequencies of PBO and *cis*- and *trans*-permethrin increased significantly over time (*p* < 0.05 controlling for potential confounders). Levels and/or detection frequencies of these compounds were significantly higher among mothers reporting use of high exposure pesticide applications (*p* ≤ 0.05). Chlorpyrifos and diazinon levels decreased significantly over time (*p* < 0.001).

**Conclusion:**

In this cohort, pest infestations, use of pesticides, and use of permethrin appear to increase after the residential restriction of organophosphorus insecticides. This is one of the first studies to document widespread residential exposure to PBO.

Understanding the pattern of residential pesticide use and exposure is critical to identifying risks and devising prevention strategies. Widespread residential pesticide use among urban communities in the United States has resulted in ubiquitous, low-level exposure to pesticides. Research documenting the potential for developmental and reproductive toxicity resulting from low levels of pesticides led to stringent and uniform pesticide regulations focusing on the safety and protection of infants and children (National Research Council 1993). This legislation, the Food Quality Protection Act (FQPA; 1996), profoundly affected the pattern of residential pesticide use in the United States. For example, in 2000–2001, the U.S. Environmental Protection Agency (EPA) withdrew the residential registrations for two commonly applied pesticides, chlorpyrifos and diazinon (U.S. EPA 2000, 2001). Before these regulations, chlorpyrifos and diazinon were the most widely used insecticides for residential pest control in the United States, including among inner-city communities in New York City (Thier et al. 1998). Subsequent studies have demonstrated the 2000–2001 EPA regulations nearly eliminated the sale of both chlorpyrifos and diazinon in these communities (Carlton et al. 2004). Data from the Columbia Center for Children’s Environmental Health (CCCEH) also show a highly significant decrease in levels of chlorpyrifos and diazinon in environmental and biologic samples collected between 1999 and 2001 from New York City African Americans and Dominicans during pregnancy, although the mothers reported no change in their use of pesticides. These initial findings indicated that the degree of pesticide use had not changed, but the mix of active ingredients contained in pesticide formulations had changed. It is important to understand which insecticides are coming into use to replace the withdrawn organophosphorus insecticides as a critical first step in evaluating whether there are any potential risks associated with the replacement insecticides, including during gestation and early childhood.

Several lines of evidence suggest that pyrethroids are replacing organophosphates for residential pest control (Bekarian et al. 2006; Whyatt et al. 2003, 2007). Pyrethroids are selected as replacement pesticides because they are potent insecticides with relatively low mammalian toxicity (Ray and Fry 2006). Low mammalian toxicity, relative to insects, is attributed to increased levels of detoxifying enzymes in mammals, higher body temperature (pyrethroids show a negative temperature coefficient of action), and an inherently lower sensitivity of the analogous mammalian ion channel target sites (Ray and Fry 2006; Song and Narahashi 1996). Additionally, pyrethroids are relatively nonvolatile, thus leading to speculation that inhalation exposure after use for residential pest control would be minimal (Pauluhn 1999). To date, pyrethroid pesticides have not been reevaluated according to the updated FQPA safety standards, and significant data gaps must be filled before a full health-hazard assessment can be completed. Like many other classes of pesticides, pyrethroids are neurotoxicants, and mechanistic and laboratory studies suggest that there is potential for developmental neurotoxicity (Shafer et al. 2005).

To our knowledge, no studies have documented an increase in pyrethroid insecticides in personal or indoor air after the restrictions on chlorpyrifos and diazinon use. This can likely be attributed to pyrethroid characteristics; most pyrethroids are infrequently detected in indoor air samples because of their low volatility (Lewis et al. 1994; Soderlund et al. 2002). To assess the use of pyrethroids for residential pest control, we propose using piperonyl butoxide (PBO) as a proxy for pyrethroid use. PBO is the leading pesticide synergist added to many residential pyrethroid formulations and can be added at 5–20 times the concentration of the active insecticidal ingredient. PBO is both more volatile and more stable in indoor air and dust than pyrethroids (Fischer and Eikmann 1996). Although PBO has been known to synergize other insecticide compounds including fipronil, parathion, and some carbamates, its current use is exclusively associated with pyrethroids (Casida 1998; Tozzi 1998); thus, detectable levels of PBO stem solely from an application of a synergized pyrethroid formulation. In a prior study of contemporary pesticides in personal air samples collected from a subset of subjects enrolled into the CCCEH cohort between 1998 and 1999, PBO was detected in over 80% of personal air samples (*n* = 71) (Whyatt et al. 2002). More recently, PBO was detected in 68.5% and 45.5% of personal and indoor air samples, respectively, from an additional subset of CCCEH cohort women (*n* = 89) enrolled during 2001–2004 (Whyatt et al. 2007).

Whereas earlier studies of this cohort have focused primarily on the most frequently detected semivolatile pesticides including chlorpyrifos, diazinon, and propoxur (Whyatt et al. 2002, 2004), the current study extends our prior research by measuring permethrin (*cis*- and *trans*-isomers) and PBO, as well as chlorpyrifos and diazinon, in personal air samples. For this study we selected samples from a larger subset of women (*n* = 511) enrolled into the cohort over an extended time frame (2000–2006). Our objective was to evaluate changes in levels of these compounds in response to the U.S. EPA regulations. The study also assessed changes in self-reported pest infestation levels and use of pest-control measures during pregnancy after the regulations. In addition, we examined the association between permethrin and PBO and self-reported pesticide use. Finally, we determined whether the decrease in chlorpyrifos and diazinon levels observed in earlier studies of personal and indoor air samples continued through 2006.

## Methods

The women included in this report are part of an ongoing prospective cohort study of African-American and Dominican women and their newborns from inner-city communities in New York City being conducted by the CCCEH. Subject enrollment, data collection, and laboratory analysis for the parent cohort study are described elsewhere (Perera et al. 2002; Whyatt et al. 2002). Briefly, we recruited women into the study during pregnancy through the pre-natal clinics at New York–Presbyterian and Harlem Hospitals. Enrollment was restricted to women 18–35 years of age who self-identified as African American or Dominican and had resided in Northern Manhattan or the South Bronx for ≥ 1 year before pregnancy. Women were excluded if they smoked cigarettes or used other tobacco products during pregnancy; used illicit drugs; had diabetes, hypertension, or known HIV infection; or had their first pre-natal visit after the 20th week of pregnancy. To date, 725 subjects have been enrolled into the parent cohort. For the current analyses, we restricted analysis to those subjects enrolled into the cohort between 2000 and 2006 based on availability of questionnaire and air monitoring data. The demographic characteristics of this subset of subjects do not differ significantly from the parent cohort. The study was approved by the Institutional Review Board of Columbia University, and informed consent was obtained from all study subjects.

### Questionnaire

A 45-min questionnaire was administered to each woman in her home by a trained research worker during the third trimester of pregnancy. It included information on demographics, home characteristics, lifetime residential history, history of active and passive smoking, occupational history, alcohol and drug use during pregnancy, housing disrepair, and history of residential pesticide use. The information on pesticide use included whether the subject sighted pests (including cockroaches, rodents, and other pests) in the home during pregnancy and the frequency of pest sightings. We also asked women whether any pest control measures were used at any time during pregnancy by a professional pesticide applicator or by others (the woman, other household members, or the building superintendent). Questions included whether the method had been used and for which type of pest it was used. If pest control measures were used, we asked about the following eight specific methods: sticky traps, bait traps, boric acid, gels, spray by a professional pesticide applicator, can sprays, pest bombs, and any other methods. General pesticide use information was available for 497 of 511 (97%) subjects.

### Forty-eight-hour personal air samples

#### Sample collection

During their third trimester of pregnancy, women in the cohort were asked to wear a small backpack holding a personal ambient air monitor during the daytime hours for 2 consecutive days and to place the monitor near the bed at night. The personal air-sampling pumps (400S pump; B.G.I., Waltham, MA) operated continuously at 4 L/min over this period and collected fine particles ( ≤ 2.5 μm in aerodynamic diameter) on a precleaned quartz microfiber filter (QMA; Whatman International, Maidstone, UK) and semivolatile vapors and aerosols on a polyurethane foam plug backup (stock #45; San Antonio Foam Fabricators, San Antonio, TX). We focused our air-monitoring methodology on the collection of fine particles, as they are considered to be of significant human health concern because of their variable composition and ability to penetrate deep into the lungs. We drew an average of 11.1 m^3^ of air through the sampler.

At the conclusion of the personal air monitoring, field staff transported the filters directly from the subject’s home back to the laboratory at the Mailman School of Public Health. Samples were immediately inventoried and frozen at approximately −15°C. Although we did not keep a record of elapsed transport time, we estimate that it was generally less than 1 hr. Quality control criteria for the air-sampling methodology have been thoroughly described (Whyatt et al. 2002).

#### Laboratory analysis

Within 1–2 months of collection, air samples were shipped on ice to Southwest Research Institute (SwRI), San Antonio, Texas, and stored at −12°C. Within 10 days of arrival at SwRI, the polyurethane foam plug and filter were placed in a Soxhlet extractor (Corning, Corning, NY), spiked with terphenyl-d_14_ as a recovery surrogate, and extracted with 6% diethyl ether in hexanes for 16 hr. The number of days elapsed between sample collection and extraction averaged 37.7 ± 2.6 days (mean ± SE). The extract was concentrated to 1 mL and frozen at −12°C before analysis. Target pesticides are stable under these conditions. We determined the amounts of the target pesticides (PBO, *cis*- and *trans*-permethrin, chlorpyrifos, and diazinon) in samples using an Agilent 6890 gas chromatograph/5973 mass spectrometer (Agilent Technologies, Palo Alto, CA) (Geno 1995; Oritz et al. 2000). We selected two ions per analyte for low-level detection and performed quantification using deuterated polyaromatic hydrocarbons as internal standards (before June 2001) or isotopically labeled standards (after June 2001) (U.S. EPA 1999). Changing the quantification method did not alter the limits of detection (LODs) for the compounds in the current analyses. The LOD for each compound was calculated as one-third of the lowest quantitation standard in the initial calibration curve. Pesticide levels less than the LOD were treated as 0.5 × LOD in the statistical analysis.

For quality assurance and quality control (QA/QC) of the analysis of pesticides in personal air samples, 10 field spikes of target pesticides (including *cis*- and *trans*-permethrin, chlorpyrifos, and diazinon) were collected along with mother’s personal air samples in 2000–2001 and extracted and analyzed in 2001. Recoveries of *cis*- and *trans*-permethrin, chlorpyrifos, and diazinon were good (> 70%) and similar to laboratory spike recoveries, indicating no degradation during sampling, processing, or handling. In addition, 13 field blanks were collected and analyzed between 2000 and 2001. Neither *cis*- nor *trans*-permethrin were detected in any of the blank samples. Chlorpyrifos and diazinon were detected in 9/13 blank samples; levels ranged between 1.02 and 3.88 ng/extract for chlorpyrifos and 1.09 and 5.04 ng/extract for diazinon. These levels were considerably lower than the levels detected in the mother’s personal air samples. PBO was not included in the personal air QA/QC samples analyzed in 2001. However, ambient indoor air-monitoring samples and field blanks were collected and analyzed between 2002 and 2004 using a method similar to the personal air monitoring. Eight indoor air field blanks were collected and analyzed. PBO was not detected in any field blanks.

## Statistical Analysis

We analyzed demographic data before main hypothesis testing. We used chi-square analyses to test whether demographic characteristics including age, ethnicity, education level, marital status, income, and receipt of public assistance differed by year of enrollment in the study. We also compared subjects enrolled in the study before the 31 December 2001 termination of retail sales of chlorpyrifos to those subjects enrolled on or after the termination date.

For analysis of residential pesticide use, we categorized the eight specific types of pesticide applications into two categories, low exposure and high exposure, based on the potential for inhalation after the applications. Low-exposure pesticide application refers to the use of boric acid, gels, bait traps, and/or sticky traps by the subject, a resident of the home, or a professional pesticide applicator. High-exposure pesticide application refers to the use of can sprays, pest bombs, and/or foggers by the subject, a resident of the home, or a professional pesticide applicator.

We employed both univariate and multivariate statistical approaches to examine the impact of the 2000–2001 U.S. EPA regulations (U.S. EPA 2000, 2001) on reported pest infestation, reported pesticide use, and pesticides measured in 48-hr personal air samples. Parametric and nonparametric designs were selected based on the distribution of variables. After log transformation, only chlorpyrifos and diazinon were normally distributed and thus were analyzed using parametric statistics. Univariate analyses compared data collected from subjects enrolled on or before the 31 December 2001 termination date with those subjects enrolled after the termination date (2000–2001 vs. 2002–2006). These analyses included chi-square tests for categorical correlations on frequency data (reported pest infestation, reported pesticide use, detection of target pesticides in air samples); Spearman’s rank test for correlations between nonparametric continuous data (ranks of PBO and *cis*- and *trans*-permethrin in personal air); Mann–Whitney *U* for comparison of nonparametric continuous data (levels of PBO and *cis*- and *trans*-permethrin in personal air); and Student’s *t*-test for comparison of parametric continuous data (levels of chlorpyrifos and diazinon in personal air). Multivariate analyses examined changes in reported pest infestation, reported pesticide use, and pesticides measured in personal air samples as a function of time elapsed in 6-month intervals. Logistic regression models were developed to examine categorical predictor variables, and linear regression models were developed to examine continuous predictor variables. Results of multivariate analyses are presented for the entire study period (2000–2006), for samples collected on or before the 31 December 2001 termination of chlorpyrifos (2000–2001) and for samples collected after 31 December 2001 (2002–2006). All multivariate analyses controlled for ethnicity and receipt of public assistance, because both of these variables change significantly by year of enrollment in the study, and were thus treated as potential confounders. For chlorpyrifos and diazinon, we used analysis of variance (ANOVA) with the least significant difference (LSD) test to examine trends over time, 2000–2006.

Finally, we examined the association between pesticides in personal air and reported pesticide use. Reported pesticide use was categorized as follows: *a*) no reported pesticide use; *b*) use of boric acid, gels, bait traps, and/or sticky traps; *c*) can sprays and/or pest bombs used with or without the lower exposure methods; and *d*) sprays by a professional pesticide applicator used with or without the lower exposure methods. Kruskal–Wallis or ANOVA analyses were applied to examine the association between pesticide levels in personal air and reported pesticide use. Chi-square analyses were used to determine the association between detection frequencies of the pesticides in personal air and the same four pesticide-use groups. Results were considered significant at *p* < 0.05.

## Results

**Figure 1:**
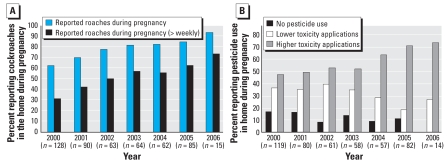
Reported cockroaches and residential pesticide use among pregnant African-American and Dominican women enrolled from prenatal clinics located in Northern Manhattan and the South Bronx between 2000 and 2006. Y/N, yes, no. Data were gathered from questionnaires administered during the 32nd week of pregnancy. (*A*) Subjects (%) reporting cockroaches in the home during pregnancy. (*B*) Subjects (%) reporting pesticide use in the home during pregnancy over time.

Table 1 provides demographic information for the 511 participants enrolled in the cohort between 2000 and 2006 for whom 48-hr personal air monitoring was completed during the third trimester of pregnancy. Demographic characteristics did not change by year of enrollment in the study except for ethnicity and receipt of public assistance. Significantly more Dominicans than African Americans were enrolled in the study after 31 December 2001 (χ ^2^ = 5.4, *p* = 0.02), and significantly more subjects reported receipt of public assistance year-by-year, 2000–2006 (χ ^2^ = 13.5, *p* = 0.04). Among this cohort, both reporting of pest sightings in the home and reported pesticide use were high and consistent with our prior results (Whyatt et al. 2002, 2003, 2007). Overall, 86% of women reported that pests (cockroaches, rats, mice and/or others) were sighted in the home during the study period. Cockroach sightings occurred most commonly (75%). As shown previously (Whyatt et al. 2002, 2003, 2005, 2007), most reported pesticide use was targeted at cockroach control. Women who reported that cockroaches were sighted in the home were significantly more likely to report use of pest control (χ ^2^ = 33.9, *p* < 0.001). Reported pest sightings and pesticide use were significantly higher among Dominican participants than African American (pest sightings: χ ^2^ = 9.2, *p* = 0.002; pesticide use: χ ^2^ = 9.9, *p* = 0.002). Eighty-eight percent of women reported using some form of pest control during pregnancy. Among these pesticide users, 32% reported using low-exposure pesticide applications such as sticky traps, bait traps, or gels, and 55% reported using one or more of the higher-exposure applications such as spray cans, bombs, or professional pesticide applicators. Reported pest infestation and pesticide use for subjects enrolled on or before 31 December 2001 and those enrolled after this date are shown in Table 2. After controlling for ethnicity and receipt of public assistance, the number of subjects reporting cockroaches in the home during pregnancy increased significantly over each 6-month interval from 63% in 2000 to 93% in 2006 {logistic regression; odds ratio (OR) = 1.14 per 6-month period [95% confidence interval (CI), 1.08–1.21]; *p* < 0.001} (Figure 1A). Further, we observed an increase in the percentage of subjects reporting cockroaches sighted in the home at least weekly [logistic regression; OR = 1.14 per 6-month period (95% CI, 1.09–1.20); *p* < 0.001]. Self-reported use of high-exposure pesticide applications (spray cans, pest bombs and/or professional pesticide applicator spray) increased over time from 47.1% in 2000 to 71.4% in 2006 [logistic regression; high-exposure pesticide applications by 6-month intervals of time elapsed; OR = 1.10 (95% CI, 1.04–1.17) controlling for ethnicity and receipt of public assistance, *p* < 0.002] (Figure 1B).

Descriptive results of pesticide detection frequencies and levels measured in personal air samples collected between years 2000 and 2006 are presented in Tables 3–5. PBO was detected in 75% of personal air samples; *cis*-and *trans*-permethrin were detected in 19% and 18% of samples, respectively. Chlorpyrifos and diazinon were detected in nearly 100% of personal air samples collected between years 2000 and 2006. Levels of *cis*-permethrin were highly correlated with *trans*-permethrin (Spearman correlation; *r* = 0.86, *p* < 0.001) and both *cis*- and *trans*-permethrin were weakly correlated with PBO (*r* = 0.29, *p* < 0.001; *r* = 0.23, *p* < 0.001, respectively). PBO measured in personal air was not associated with either chlorpyrifos or diazinon (Pearson correlation, *p* > 0.05).

Table 4 displays the detection frequencies and mean ranks of PBO, *cis*-, and *trans*- per-methrin in 48-hr personal air samples. Subjects were stratified into two groups: those enrolled before and those enrolled on or after the 31 December 2001 termination of retail sales of chlorpyrifos. Detections of *cis*- and *trans*-permethrin in personal air were higher among samples collected after the termination of organophosphate use compared with those collected before the termination date. This trend was significant for *cis*-permethrin (χ ^2^ = 12.4, *p* < 0.01). Mean ranks of both *cis*- and *trans*-permethrin were significantly higher among samples collected on or after 31 December 2001 than those collected before 31 December 2001. *cis*-Permethrin mean ranks increased from 217.3 to 270.2 (Mann–Whitney *U*, *p* < 0.001) and *trans*-permethrin mean ranks increased from 209.2 to 271.2 (Mann–Whitney *U*, *p* < 0.001). By univariate analysis, detection frequency and mean rank of PBO did not change significantly after the 31 December 2001 termination of chlorpyrifos.

Table 5 shows the detection frequencies and geometric mean (GM) (95% CI) of chlorpyrifos and diazinon in personal air samples. Detection frequencies of these compounds were consistently high throughout the study period and did not change significantly when compared before and after the 31 December 2001 termination date (*p* < 0.05). For chlorpyrifos, χ ^2^ = 1.12, *p* = 0.29; for diazinon, χ ^2^ = 0.76, *p* = 0.38. As shown in prior studies, levels of chlorpyrifos and diazinon were significantly lower among those samples collected after the implementation of the 2000–2001 regulations when compared with those collected before the 31 December 2001 termination date (Student *t*-test, *p* < 0.001).

**Figure 2:**
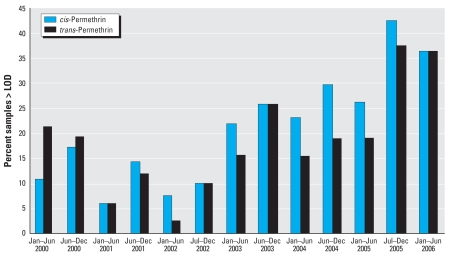
*cis*-and *trans*-Permethrin in 48-hr personal air samples collected from pregnant African-American and Dominican subjects enrolled from prenatal clinics located in Northern Manhattan and the South Bronx between 2000 and 2006.

We used regression models to determine whether the number of 6-month periods elapsed since the 31 December 2001 U.S EPA regulations (U.S. EPA 2000) significantly predicted detection frequency and levels of compounds measured in personal air samples (Table 6). After controlling for ethnicity and receipt of public aid, detections of *cis*- and *trans*-permethrin in personal air increased markedly throughout the study period [logistic regression for 2000–2006; OR = 1.17 per 6-month period (95% CI, 1.10–1.25, *p* < 0.001) and 1.09 per 6-month period (95% CI, 1.03–1.16, *p* = 0.006), respectively] (Table 6 and Figure 2). The increase in detection was stronger among samples collected after the termination of residential uses of organophosphorus insecticides [2002–2006; OR = 1.27 per 6-month period (95% CI, 1.13–1.43, *p* < 0.001) and 1.30 per 6-month period (95% CI, 1.14–1.49, *p* < 0.001), respectively] compared with those collected before the termination [2000–2001; OR = 0.77 (95% CI, 0.53–1.14, *p* > 0.05) and 1.37 (95% CI, 0.29–6.45, *p* > 0.05)]. The association between the time period and the detection frequency was significantly stronger in the period after the organophosphorus insecticide restrictions (*p* < 0.05). Detection frequency of PBO also increased after the termination of residential organophosphorus insecticide use [logistic regression; OR = 1.14 per 6-month period (95% CI, 1.02–1.27)]. As described in prior studies, levels of chlorpyrifos and diazinon in 48-hr personal air samples decreased significantly over time (linear regression, β = −0.147 per 6-month period, *p* = < 0.001; β = −0.255 per 6-month period, *p* < 0.001, respectively). However, levels for samples collected in 2005 and 2006 were not statistically different from those collected in 2004 (ANOVA LSD mean difference: 0.41, *p*=0.88; 0.51, *p*=0.72, respectively).

**Figure 3:**
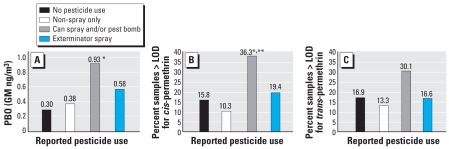
Insecticides measured in personal air samples and reported pesticide use. Personal air samples (48-hr) collected during the third trimester of pregnancy among women reporting that no pest control methods were used as well as the three following groups: use of lower-exposure pesticide applications only (non-spray); use of spray cans and pest bombs (with or without the use of the lower-exposure pesticide applications); and use of spray by a professional pesticide applicator (with or without the use of the other methods). Analysis included 309 women with air monitoring results for whom questionnaire data on use of pest control methods in the home was also collected. (*A*) Concentration of PBO (ng/m^3^). Prevalence of detection (%) of *cis*-permethrin (*B*) and *trans*-permethrin (*C*) in 48-hr personal air among different pesticide use categories. *Kruskal–Wallis, *p* < 0.001. **Chi-square test, *p* < 0.05.

Figure 3A presents the GM PBO levels in personal air samples by self-reported pesticide use group. Figures 3B and 3C show the percent of samples greater than the LOD for *cis*-and *trans*-permethrin by self-reported pesticide use group. Forty-eight-hour personal air levels of PBO were significantly higher among women reporting use of spray cans and/or pest bombs after controlling for the year of air monitoring (0.93 ng/m^3^ vs. 0.30 ng/m^3^, respectively) (Kruskal–Wallis, *p* < 0.001) (Figure 2A). Detection frequency of PBO was also higher among spray can/pest bomb users but was not statistically significant (χ ^2^ = 6.4, *p* = 0.09). Mean ranks of *cis*- and *trans*-permethrin were higher among spray can/pest bomb users, and this difference was statistically significant for *cis-*permethrin and of borderline significance for *trans*-permethrin (Kruskal–Wallis, *p* = 0.007, *p* = 0.07, respectively). Detection frequencies of *cis*- and *trans*-permethrin were also significantly greater among subjects reporting the use of spray cans/pest bombs during pregnancy (χ ^2^ = 14.3, *p* < 0.001, and χ ^2^ = 3.8, *p* = 0.05, respectively) (Figure 3B and 3C). Chlorpyrifos and diazinon were not associated with self-reported pesticide use (ANOVA, *p* > 0.05).

## Discussion

This study expands on previous work documenting widespread residential pesticide use and exposure among African-American and Dominican women residing in New York City. Whereas earlier studies of this cohort have focused primarily on the most frequently detected semivolatile pesticides including chlorpyrifos, diazinon, and propoxur (Whyatt et al. 2002, 2004), this study focused on permethrin, a pyrethroid pesticide, and PBO, a commonly used insecticide synergist in residential pyrethroid formulations. Our objectives were to determine whether the mix of active ingredients contained in insecticide formulations for residential pest control is changing over time and whether the use of per-methrin and PBO for residential pest control has increased after the U.S. EPA restrictions on residential applications of chlorpyrifos and diazinon.

We first cross-sectionally examined whether the frequency of reported pest infestations and pesticide use patterns among women enrolled into the cohort have changed over time. Despite warnings against the use of residential pesticides during pregnancy, 88% of subjects reported using pesticides at some point during pregnancy. Contrary to our expectations, we observed a significant trend toward increasing reports of pest sightings in the home and increasing use of the higher-exposure methods (spray cans, pest bombs, and/or sprays by professional pesticide applicators) over time. Our particular interest in the use of higher-exposure pesticide applications stems from our earlier investigations of pesticide products sold in the study catchment areas. According to our studies, organophosphorus and pyrethroid pesticides (with or without PBO) are largely applied through high-exposure spray cans and pest bomb applications. Increased high-exposure pesticide application and concurrent increases in cockroach sightings suggest the possibility that replacement insecticides may be less effective at controlling cockroaches and other pests than organophosphorus insecticides. Frequent use of pesticides to control insects has been known to result in insect resistance and resulting control failures (Atkinson et al. 1991; Carlton et al. 2004; Cochran 1989; Scott et al. 1990). Because of intensive use of pyrethroids for arthropod control, many arthropod populations, including cockroaches, have developed resistance to these compounds (Tan et al. 2005).

Although pesticide resistance is a likely explanation for the observed trends of increasing pest sightings and reported pesticide use among subjects in this cohort, alternative explanations are possible and should be investigated. For example, our work and that of others have clearly shown associations between the degree of housing disrepair and both pest sighting and use of pest control measures (Bradman et al. 2005; Fenske et al. 2005; Whyatt et al. 2002). In the current study, housing disrepair was assessed in the prenatal questionnaire as a sum of five factors: holes in ceilings/walls, existence of unrepaired water damage, mold, leaking pipes, and peeling/chipping paint. Among subjects in this cohort, reported housing disrepair did not change over time between years 2000 and 2006. It is possible that our assessment of housing disrepair was not sensitive enough to detect changes in housing conditions that could partially explain the significant increase in reported pests and pesticide use.

Next, we evaluated the change in personal air levels of permethrin and PBO among women enrolled before and after the 2000–2001 U.S. EPA regulations restricting the residential use of chlorpyrifos and diazinon to determine whether use of pyrethroids is increasing over time. Our data on PBO and permethrin exposure over time indicate that pyrethroid use is increasing to some extent. We first compared detection frequencies and mean ranks of PBO and permethrin from samples collected before the 2000–2001 U.S. EPA regulations to those samples collected after the regulations. Although we observed a significant increase in the mean ranks and detection frequency of both permethrin isomers after the regulations, we did not see any significant change in PBO exposure. Subsequently, we examined changes in PBO and permethrin in 6-month intervals after the 31 December 2001 withdrawal of chlorpyrifos. For all three compounds, we observed a significant increase in the likelihood of detection within each 6-month interval after the U.S. EPA regulations. Interestingly, for all three compounds, we observed a trend of decreased pesticide detection frequency and mean rank within the first 6-month interval after 31 December 2001. This trend is depicted for *cis*-permethrin in Figure 2. After this dip, pesticide exposure appears to stabilize and subsequently increase. This pattern may explain the discrepancy between the univariate and multivariate PBO analysis. It is possible that increased awareness of the risks of pesticide use generated by the 2000–2001 U.S. EPA pesticide regulations resulted in a short-term decrease in residential pesticide use. Although this trend was not reflected in our questionnaire data, a short-term decrease in pesticide use may explain why we did not see a significant increase in PBO in personal air evaluated by univariate analysis while we observed a significant increase when examined as 6-month intervals.

Levels of PBO and detection of permethrin in the maternal personal air samples were highly associated with maternal self-reported use of spray cans and pest bombs during pregnancy. As exposure to PBO is almost exclusively through the use of pyrethroid insecticides, this indicates pyrethroids are used for residential pest control among women in the cohort and use appears to be increasing. Air levels of PBO were only weakly correlated with air levels of either the *cis*- or *trans*-isomers of permethrin. These weak correlations are to be expected, as permethrin is nonvolatile and sequestered out of the indoor air environment rapidly after application (Fischer and Eikmann 1996; Pauluhn 1999), and pyrethroids other than permethrin can be formulated with PBO. However, the observed correlation between permethrin and PBO does suggest that some PBO exposure is accounted for by use of permethrin.

Our initial interest in PBO stemmed largely from its utility as a proxy for exposure to pyrethroids; however, evidence of ubiquitous and increasing exposure in our cohort of pregnant women renewed our interest in potential adverse health effects associated with prenatal and early childhood exposure. Concern for human health effects after residential exposure to PBO arises not from its intrinsic toxicity, which is relatively low (Breathnach 1998), but from its potential to interfere with P450 activity and alter the metabolism of other xenobiotic compounds, including pharmaceutical medications (Franklin 1976). Alteration of normal P450 activity in humans can impact drug elimination, leading to pharmacokinetic interactions with coadministered drugs, and may also significantly affect disease pathogenesis (Murray 2006). Studies of occupational and consumer exposure to PBO have not reported any significant effects on human health (Phillips et al. 1997), but data are limited. To our knowledge, no published studies have examined the biologic dose of PBO or resulting health effects after low-level chronic inhalation exposure in either animal models or humans. In the current study, PBO was detected in 75% of personal air samples collected during the third trimester of pregnancy between 2000 and 2006 from African-American and Dominican women residing in New York City, and exposures were generally low (0.19–104.86 ng/m^3^). Given our evidence of widespread exposure to PBO during pregnancy, further investigation into the potential of PBO to alter the metabolism of other xenobiotic compounds appears warranted.

Finally, we evaluated the change in chlorpyrifos and diazinon in maternal personal air samples collected throughout 2000–2006. Previously, we detected a significant decrease in levels of both compounds in personal air samples collected between 1998 and 2002 and in a small subset of personal and indoor air samples collected between 2001 and 2004 (Whyatt et al. 2005, 2007). The current study follows a larger group of women enrolled over an extended time frame. Consistent with our prior data, we observed a highly significant decrease in maternal personal air levels of chlorpyrifos and diazinon among women enrolled in 2000–2006. This decrease appears to level off after 2004. Nonetheless, chlorpyrifos and diazinon are still detected in 92% of personal air samples collected in 2006, up to 5 and 4 years, respectively, after the restriction of their residential use. This suggests that both compounds are quite persistent in the indoor environment. From prior work completed at the CCCEH (Carlton et al. 2004), we know that the U.S. EPA restrictions on organophosphorus compounds were effective at eliminating sales of these compounds for residential pest control. By 2003, 1 year after the restriction of diazinon and 2 years after the phase-out of chlorpyrifos, a survey of 109 stores in the catchment area of the CCCEH study showed that only one store sold products containing chlorpyrifos, and 18% (*n* = 20) of stores sold products containing diazinon (Carlton et al. 2004).

Limitations of the study should be noted. Information regarding the type and frequency of pesticides used for residential pest control was gathered from questionnaires administered to subjects during the third trimester of pregnancy. We asked subjects whether pesticides were used in the home during pregnancy and, if so, the frequency of use (> 1 time/week, 1 time/week, 1–3 times/month, once a month, < once a month). However, as we did not ask about the last date of pesticide use, we do not have information regarding the time elapsed between the most recent pesticide application and the occurrence of the personal air monitoring. In addition, although we asked subjects about the product brand names used for residential pest control, women were frequently unable to supply these data. In preliminary analyses of questionnaires, women provided a pesticide product name for only 39% of the pest control methods reported to be used in the home during pregnancy and, in particular, were rarely able to identify the pesticide products used by an exterminator. Further, pesticide products can have the same brand name but contain different active ingredients (Fenske et al. 2005).

An addition limitation of the study design involves the environmental sampling methodology. We noted that the infrequent detection of permethrin in personal air was expected. Permethrin is not volatile and may be better monitored in dust than air. The CCCEH Mothers and Newborns Study was initiated in 1998 when the primary pesticides used for residential pest control were semivolatile. Accordingly, the current sampling methodology was designed to measure pesticides in air. Because of funding limitations, we were unable to modify our sampling methodology to include dust. However, nonvolatile permethrin is clearly detectable in air as a result of recurrent pesticide use (Whyatt et al. 2007) and through resuspension of dust particles. In addition, our current data clearly show a significant increase in permethrin consistent with increased use.

Collectively, these findings indicate that the mix of pesticides being used for residential pest control has changed and that pyrethroids are replacing the organophosphorus insecticides for residential pest control among this cohort. Several other lines of evidence suggest that the mix of compounds used for residential pest control is changing in response to the restricted use of organophosphate pesticides. These include *a*) recommendations by the U.S. EPA of alternatives to chlorpyrifos and diazinon for home use (U.S. EPA 2002); *b*) a survey by the attorney general of New York State of pesticides used by residents of public housing (Surgan et al. 2002); and *c*) point-of-sale tracking of indoor-use permethrin products indicating a 15% increase between 1997 and 2002 (Bekarian et al. 2006). Research is ongoing in the current cohort to measure levels of the pyrethroids in biologic samples (urine and plasma) collected from the mother during pregnancy and from the mothers and newborns at delivery and to determine whether residential use of pyrethroids has resulted in internal doses to the mother and fetus during pregnancy. Exploratory analyses will examine the association between prenatal exposure to pyrethroids and PBO and the potential for adverse neurodevelopmental outcomes.

## Figures and Tables

**Table 1:** Demographic characteristics (%) for African-American and Dominican subjects enrolled during pregnancy from Northern Manhattan and the South Bronx throughout 2000–2006 (*n* = 511).

Characteristic	2000–2001	2002–2006
No. of subjects^a^	219	292
Age (years)^b^	24.1 (18–38)	25.8 (18–36)
Ethnicity		
African American	37.4*	27.7*
Dominican	62.6*	72.3*
Education		
< High school degree	36.7	38.0
High school degree or equivalent	37.2	36.3
> High school degree	26.1	25.7
Marital status^c^		
Never married	68.7	60.8
Married	31.3	39.2
Income		
< $10,000/year	39.3	44.7
> $10,000/year	60.7	55.3
Receiving public assistance^d^	91.7*	92.1*
Housing disrepair^e^	59.6	63.2

Subjects are grouped as those enrolled before and those enrolled on or later than the 31 December 2001 termination of all retail sales of chlorpyrifos.

a Missing values: education (1), income (41), housing disrepair (2).

b Mean (range).

c Includes women living as married with same partner > 7 years.

d Includes receipt of Medicaid and/or federal aid.

e Estimate of housing disrepair as a sum of five factors reported in questionnaire: inability to afford electricity, unrepaired water damage, mold, leaking pipes, and peeling or chipping paint.

* Chi-square test, *p* < 0.05.

**Table 2:** Subjects (%) reporting pest sightings and use of pest control measures in the home during pregnancy among a cohort of African-American and Dominican subjects living in Northern Manhattan and the South Bronx throughout years 2000–2006 (*n* = 511^a^).

Characteristic	2000–2001 (*n* = 219)	2002–2006 (*n* = 292)
Total reporting pest sightings	79.4	91.4*
Cockroaches	65.6	82.4*
Reported cockroaches at least weekly/daily	35.8	57.6**
Rodents	51.4	56.6
Reported rodents at least weekly/daily	41.3	63.3**
Total reporting use of pest control measures	84.7	90.4
Low-exposure application only^b^	32.9	28.1
High-exposure application	47.7	61.2*
Can sprays and/or pest bombs	16.3	28.2*
Professional pesticide applicator sprays	41.0	39.6

Subjects are grouped as those enrolled before and those enrolled on or later than the 31 December 2001 termination of all retail sales of chlorpyrifos.

a Missing values: cockroaches (4), rodents (3), pest control measures (14).

b Sticky traps, bait traps, boric acid, or gels.

* Chi-square test, *p* < 0.05.

** Chi-square test, *p* < 0.001.

**Table 3:** Concentrations of chlorpyrifos, diazinon, permethrins, and PBO in 48-hr personal air samples collected during the third trimester of pregnancy from 511^a^ African-American and Dominican women enrolled between 2000 and 2006 from prenatal clinics located in Northern Manhattan and the South Bronx.

			Percentile (%)				
Pesticide	LOD (ng/m^3^)^b^	No. > LOD (%)	25	50	75	95	GM (95% CI)^c^
Chlorpyrifos	0.10	494 (99.0)	1.44	2.78	6.58	25.96	3.2 (2.9–3.6)
Diazinon	0.10	497 (99.8)	3.21	8.22	22.28	194.35	14.7 (12.8–16.7)
*cis*-Permethrin	0.18	96 (19.4)	< LOD	< LOD	< LOD	1.51	NC
*trans*-Permethrin	0.36	87 (17.8)	< LOD	< LOD	< LOD	2.30	NC
PBO	0.10	254 (75.3)	0.19	0.42	1.19	12.15	0.54 (0.5–0.6)

Abbreviations: GM, geometric mean; NC, not calculated.

a Missing values: chlorpyrifos (13), diazinon (14), *cis*-permethrin (16), *trans*-permethrin (21), PBO (175).

b LODs were determined as nanograms per extract. On calculation of nanograms per cubic meter, LODs varied slightly, depending on the concentration of the extract and the amount of air samples over 48 hr. Mean volume of air sample: 11.1 ± 0.6 m^3^.

c GM was calculated only if pesticide was detected in > 50% of samples; levels in samples without detections were set at one-half the detection limit.

**Table 4:** Change in detection frequency and concentration of *cis*- and *trans*-permethrin and PBO in 48-hr personal air samples collected before versus on or after the 31 December 2001^a^ termination of retail sales of chlorpyrifos.

	2000–2001 (*n* = 219)^b^		2002–2006 (*n* = 292)^b^			
	% > LOD	Mean rank	% > LOD	Mean rank	Chi-square^c^	MWU^d^
*cis*-Permethrin	12.2	217.3	24.8	270.2	12.4 (*p* < 0.01)	*p* < 0.001
*trans*-Permethrin	15.4	209.2	19.5	271.3	1.4 (*p* = 0.24)	*p* < 0.001
PBO	77.8	180.06	74.8	166.29	0.21 (*p* = 0.64)	*p* = 0.340

Samples were collected during the third trimester of pregnancy from African-American and Dominican women living in Northern Manhattan and the South Bronx (*n* = 511).

a Date selected based on U.S. EPA deadline to terminate all sales of chlorpyrifos for residential use by 31 December 2001. Similar restrictions curtailed the use and sales of diazinon by 31 December 2002.

b Missing values 2000–2001: PBO (175), *cis*-permethrin (16), *trans*-permethrin (21); 2002–2006: PBO (10).

c Chi-square test used to compare detection frequency of compounds among samples collected before versus on or after 31 December 2001.

d Mann–Whitney *U* used to compare mean rank concentration of compounds among samples collected before versus on or after 31 December 2001.

**Table 5:** Change in concentration [GM (95% CI)] of chlorpyrifos and diazinon in 48-hr personal air samples collected before versus on or after the 31 December 2001^a^ termination of retail sales of chlorpyrifos.

	2000–2001 (*n* = 219)^b^		2002–2006 (*n* = 292)^b^		
	Percent > LOD	GM	Percent > LOD	GM	Student *t*-test^c^
Chlorpyrifos	99.5	5.2 (4.4–6.2)	98.6	2.3 (2.0–2.6)	*p* < 0.001
Diazinon	100	23.3 (20.1–26.5)	99.6	4.3 (3.7–5.1)	*p* < 0.001

Samples were collected during the third trimester of pregnancy from African-American and Dominican women living in Northern Manhattan and the South Bronx (*n* = 511).

a Date selected based on U.S. EPA deadline to terminate all sales of chlorpyrifos for residential use by 31 December 2001. Similar restrictions curtailed the use and sales of diazinon by 31 December 2002.

b Missing values 2000–2001: chlorpyrifos (13), diazinon (14).

c Student *t*-test used to compare log-transformed concentration of compounds detected in 48-hr personal air before versus on or after 31 December 2001.

**Table 6:** Logistic and linear regression models for target compounds measured in 48-hr personal air samples collected during the third trimester of pregnancy from African-American and Dominican women living in Northern Manhattan and the South Bronx (*n* = 511).

Model^b^	2000–2006^a^ (*n* = 511)	2000–2001^a^ (*n* = 219)	2002–2006^a^ (*n* = 292)
Logistic regression^c^			
*cis*-Permethrin	1.17 (1.10–1.25)*	1.13 (0.75–1.68)	1.27 (1.13–1.43)
*trans*-Permethrin	1.09 (1.03–1.16)*	0.77 (0.53–1.14)	1.30 (1.14–1.50)
PBO	1.07 (0.98–1.16)	1.37 (0.29–6.45)	1.14 (1.02–1.27)
Linear regression^d^			
Chlorpyrifos	−0.147 (< 0.001)*	−0.056 (0.519)	−0.146 (< 0.001)
Diazinon	−0.255 (< 0.001)*	−0.148 (0.086)	−0.273 (< 0.001)

Regression models were used to determine whether the number of months (as 6-month intervals) elapsed since the 31 December 2001 U.S. EPA regulations predicted the detection frequency and levels.

a Missing values 2000–2006: PBO (177), *cis*-permethrin (18), *trans*-permethrin (18), chlorpyrifos (13), diazinon (14); 2000–2001: PBO (166), *cis*-permethrin (7), *trans*-permethrin (7), chlorpyrifos (3), diazinon (4); PBO (11), *cis*-permethrin (11), *trans*-permethrin (11), chlorpyrifos (10), diazinon (11); 2002–2006: PBO (10), *cis*-permethrin (10), *trans*-permethrin (10), chlorpyrifos (10), diazinon (10).

b Models control for ethnicity (Dominican = 0, African American = 1) and receipt of public assistance (no = 0, yes = 1).

c OR from logistic regression represents pesticide detection as a function of time elapsed in 6-month intervals.

d β (*p*-value) from linear regression represents log- transformed pesticide concentrations as a function of time elapsed in 6-month intervals.

* Significant interaction term, *p* < 0.01.
